# New Organic/Inorganic Pigments Based on Azo Dye and Aluminum-Magnesium Hydroxycarbonates with Various Mg/Al Ratios

**DOI:** 10.3390/ma12081349

**Published:** 2019-04-25

**Authors:** Anna Marzec, Bolesław Szadkowski, Jacek Rogowski, Waldemar Maniukiewicz, Przemysław Rybiński, Mirosława Prochoń

**Affiliations:** 1 Institute of Polymer and Dye Technology, Faculty of Chemistry, Lodz University of Technology, Stefanowskiego 12/16, 90-924 Lodz, Poland; boleslaw.szadkowski@edu.p.lodz.pl (B.S.); miroslawa.prochon@p.lodz.pl (M.P.); 2 Institute of General and Ecological Chemistry, Lodz University of Technology, Zeromskiego 116, 90-924 Lodz, Poland; jacek.rogowski@p.lodz.pl (J.R.); waldemar.maniukiewicz@p.lodz.pl (W.M.); 3 Department of Management and Environmental Protection, Jan Kochanowski University, Zeromskiego 5, 25-369 Kielce, Poland; przemyslaw.rybinski@ujk.edu.pl

**Keywords:** hybrid materials, azo chromophore, pigment characterization, polymer composite

## Abstract

This study set out to investigate the impact of aluminum-magnesium hydroxycarbonates (LHs) with various Mg/Al ratios on the formation of hybrid pigments. The colorants were also evaluated for their flame-retardant properties. In the first part of the study, the hybrid pigments were submitted to comprehensive characterization using time-of-flight secondary ion mass spectrometry (TOF-SIMS), ^27^Al solid-state nuclear magnetic resonance (NMR) spectroscopy, powder X-ray diffraction analysis (XRD), thermogravimetric analysis (TGA), and N_2_ adsorption as well as scanning and transmission electron microscopy (SEM/STEM). The relationship between the Mg/Al ratios of the LH carriers and the formation of lake pigments was explored. The TOF-SIMS spectrum of LH modified with azo chromophore (AC) showed an intense peak for the C_19_H_15_O_5_N_2_Mg^+^ ion, confirming metal-dye interactions. Incorporation of the organic colorant into the LH host enhanced its resistance to dissolution in organic solvent (butyl acetate), as well as improving its color stability under elevated temperatures. The second part of the study evaluated the flammability of ethylene-norbornene (EN) composites, in which the pigments had been applied as colorants. Cone calorimetry revealed that addition of the organic-inorganic pigments resulted in a substantial improvement of the flame retardancy, reflected by the decreased values of the heat release rate (HRR_MAX_) and total heat release parameter (THR) of the EN composites in comparison to a neat sample (unfilled EN).

## 1. Introduction

Organic pigments spread throughout the medium to be colored in the form of crystalline particles. The final properties of pigmented materials depend, therefore, not only on the molecular structure of the organic pigment, but also, crucially, on its crystal structure [1,2]. The structures of hybrid materials obtained by the combination of organic dyes with inorganic substrates have been the subject of extensive research, due to their desirable optical attributes such as brightness and/or fluorescence, and important properties such as chemical, thermal and light stability [3,4,5,6]. Numerous studies have reported the stabilization and immobilization of organic dyes on different inorganic bases, including zeolites, gamma-alumina, layered double hydroxides, silica, and clay minerals, producing pigments with enhanced applicative performance (insolubility, chemical and thermal resistance) [7,8,9,10,11,12,13,14,15,16]. Such hybrid pigments, when applied to polymers, can act as multifunctional additives that modify different properties of the materials (transparency, mechanical properties or thermal stability). The modification of inorganic carriers inspired by the ancient Maya Blue pigment has been found to be a promising way of transforming soluble natural and synthetic dyes into insoluble organic-inorganic pigments [17,18,19,20,21,22]. Inorganic hosts, including aluminum hydroxide (Al(OH)_3_), magnesium hydroxide (Mg(OH)_2_) or layered double hydroxides, are commonly used in technology, and represent a large group of halogen-free flame retardants for polymer materials [23,24,25]. During combustion, a protective inorganic-rich layer forms on the sample surface, which contributes to enhancing its fire resistance [26,27].

Many studies have researched the influence of metal hydroxides, unmodified or intercalated layered double hydroxides, on the different properties of composites. However, there is little in the literature on the application of dye-modified mixed oxides in polymer materials. Kang et al. [28] synthesized structure-intercalated layered double hydroxide, which they applied as a novel functional fire-retarding nanofiller for polypropylene-grafted maleic anhydride composite. Marangoni et al. [29,30] used layered double hydroxide intercalated with different dye anions to produce transparent, colored, poly(vinyl chloride) films with improved mechanical performance. Such intercalated/adsorbed organic/inorganic and stabilized materials have great potential as colored fillers for polymer composites. 

In our previous work, we studied the effect of dye types and their functionalities on the properties of lake pigments. Azo and anthraquinone chromophores were precipitated mainly by aluminum-magnesium hydroxycarbonate with Mg/Al ratios of 30:70 [31,32]. However, the nature and morphology of the applied carrier may also affect the formation of organic/inorganic pigments. We obtained good results for aluminum-magnesium hydroxycarbonate when the concentration of magnesium was lower than in the case of traditional layered double hydroxides. Therefore, in our current study we decided to study the influence of aluminum-magnesium hydroxycarbonate characterized by different Mg/Al ratios on the formation of organic/inorganic pigments.

The present study set out to investigate the effect of dye-modified aluminum-magnesium hydroxycarbonate (hereafter LH) compounds with various Mg/Al ratios on the flammability of ethylene-norbornene (EN) composites. In the first stage, a simple one-step precipitation method was used to produce organic-inorganic pigments. Four different aluminum-magnesium hydroxycarbonates (Mg/Al weight ratio 5/95; 20/80; 30/70; 70/30) were used as the inorganic hosts. The physical and chemical properties of the pigments were studied for each carrier type, using various techniques including powder X-ray diffraction analysis (PXRD), thermogravimetric analysis (TGA) as well as scanning and transmission electron microscopy (SEM/STEM). The flammability of the EN/hybrid pigment compositions was studied by cone calorimetry.

## 2. Materials

**Figure 1:**
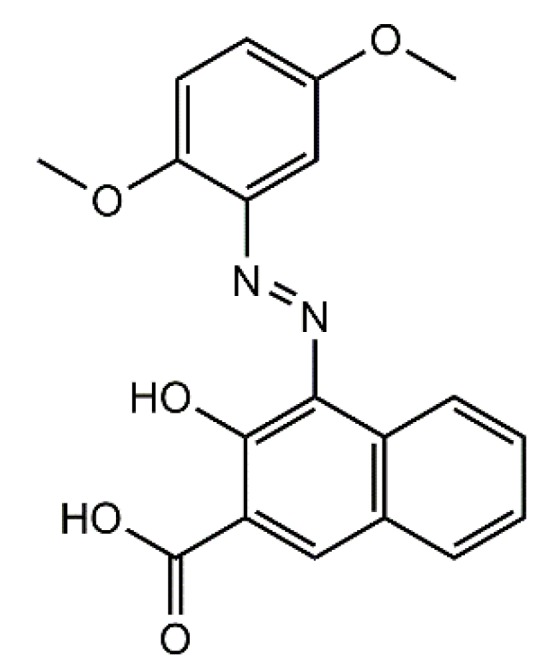
Chemical formula of carboxylic azo chromophore (AC).

Aluminum-magnesium hydroxycarbonates (hereafter designated as LHs) with various Mg/Al ratios (5/95; 20/80; 30/70; 70/30) were provided by the Sasol GMbH company (Hamburg, Germany). The azo chromophore, hereafter designated as AC (Figure 1), was prepared following the typical diazotization procedure [33]. The substrates for AC synthesis, 2,5-dimethoxyaniline (98%), 3-hydroxy-2-naphthoic acid and hydrochloric acid (37%), as well as other organic solvents (acetic acid, butyl acetate, ethyl alcohol), all analytical grade, were purchased from Sigma-Aldrich (St. Louis, MO, USA).

### Hybrid Pigment Preparation

**Figure 2:**
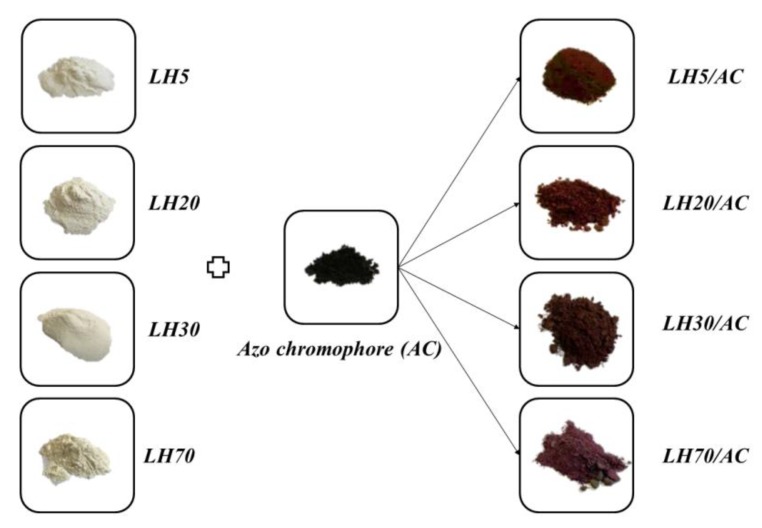
Digital photos of studied powder samples.

The hybrid pigments were synthesized via the incorporation of a specific amount of azo chromophore into the LH host. In order to obtain LH/AC hybrids with dye concentrations of 15%, 1.5 g of the synthetic azo dye was dissolved in 200 mL of deionized water with 15 mL of ethanol. The mixture was sonicated for 30 min, then heated to 80 °C, at which point 8 g of LH support was added. The dispersion was stirred for 3 h, after which the mixture was filtered off under reduced pressure. Then, the obtained mass was washed several times with water until a colorless filtrate was observed. Finally, the sediment was dried in an oven at 70 °C for the next 24 h under static air atmosphere to obtain dry powder pigment. In this way, different shades of hybrid pigments were synthesized (Figure 2).

Figure 2 shows digital images of the prepared hybrid pigments. As can be seen, modification of the different LHs with AC resulted in burgundy-like color pigments. The shade of hybrid pigments changed as the Mg/Al weight ratio in LH matrix increased from 5:95 to 70:30.

## 3. Characterization

In the present study, secondary ion mass spectrometry was performed using a TOF-SIMS IV mass spectrometer (Ion-Tof GmbH, Münster, Germany) equipped with high mass resolution time of flight mass analyzer. The measurement area covered 100 μm × 100 μm of the sample surface and the analysis time was 30 s. The structure of the materials was studied by X-ray powder diffraction analysis using a PANalytical X’Pert Pro MPD diffractometer in the Bragg-Brentano reflecting geometry, equipped with a CuKα radiation source (Malvern Panalytical Ltd., Royston, UK). Measurements were performed in the range of *2θ* = 2–70° with a step size of 0.0167°. The morphology of the powder samples was investigated using a LEO 1350 Gemini Scanning Electron Microscope (Zeiss/LEO, Oberkochen, Germany) and a high-resolution Scanning Transmission Electron Microscope (FEI, NovaNanoSEM 450, Waltham, MA, USA; accelerating voltage 30 kV), for SEM and STEM analyses, respectively. The samples for STEM measurement were prepared by depositing the colloids onto carbon-coated copper grids. The thermal stability of the hybrid pigments was evaluated by thermogravimetric analysis (TGA/DTG) using a TA Instruments Q500 Thermogravimetric Analyzer (TA Instrument, Greifensee, Switzerand). Samples of approximately 5 mg were placed in aluminum pans and heated from 25 to 600 °C with a heating rate of 10 °C/min. The specific surface area was determined based on nitrogen adsorption experiment at 77 K (Micromeritics, Norcross, GA, USA) according to the Brunauer-Emmett-Teller (BET) nitrogen adsorption method. Prior to the adsorption experiment, powder samples were degassed for 20 h at 100 °C under a vacuum. The solvent resistance of the hybrid pigments was determined in accordance with the PN-C-04406/1998 standard. Small amounts (about 0.05 g) of the pigments were immersed in butyl acetate for 24 h. The degree of decolorization was evaluated after 24 h. Elemental analysis was performed on a Vario EL III analyzer (Elementar Analysensysteme GmbH, Langenselbold, Germany). The color of the hybrid pigments was measured using a CM-3600d spectrophotometer (Konica Minolata Sensing Inc., Osaka, Japan). The spectral range for the measurements was 360–740 nm and the total color change (*ΔE*) was calculated using the formula:

(1) $$\Delta E=\sqrt{{\left(\Delta L\right)}^{2}+{\left(\Delta a\right)}^{2}+{\left(\Delta b\right)}^{2}}$$

where Δ*L* is the level of lightness or darkness, Δ*a* is the relationship between redness and greenness, and Δ*b* is the relationship between blueness and yellowness.

The polymer composites were prepared by mixing ethylene-norbornene copolymer (100 phr—parts per hundred parts of rubber) with synthesized hybrid pigments (5 phr) using a Brabender laboratory-scale measuring mixer N50. The mixing process was carried out with the following parameters: rotor speed of 50 rpm, temperature of 110 °C. The flammability of the resulting EN compounds was examined using a cone calorimeter (Fire Testing Technology Ltd., East Grinstead, UK) according to the PN-ISO 5600 standard. In this test, squared specimens (100 mm × 100 mm × 2 mm) were irradiated horizontally with a heat flux of 35 kW/m^2^.

## 4. Results and Discussion

### 4.1. Time-of-Flight Secondary Ion Mass Spectrometry (TOF-SIMS)

**Figure 3:**
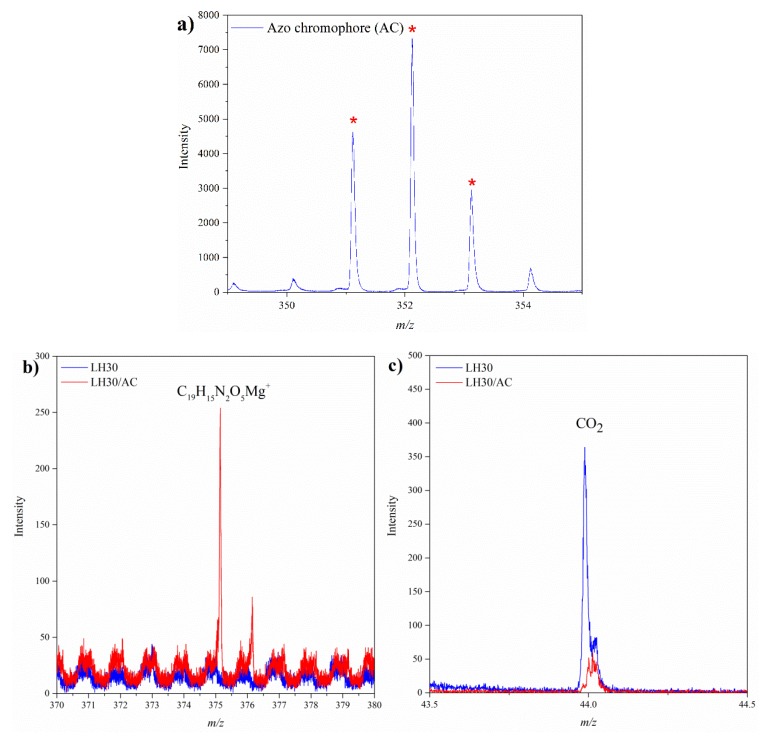
Time-of-flight secondary ion mass spectrometry TOF-SIMS spectra of AC dye (**a**), LH30 (**b**) and LH/AC samples, negative secondary CO_2_^−^ ions in LH30 and LH30/AC (**c**).

Time-of-flight secondary ion mass spectrometry was used to investigate the interactions between the LH host and the azo chromophore. This technique has previously been applied successfully to the study of interactions between the anthraquinone chromophore and Al and Mg ions from the LH matrix [32]. TOF-SIMS analysis of the uncompleted dye showed efficient formation of C_19_H_16_O_5_N_2_^+^ molecular ions from the AC dye (Figure 3a), in agreement with their isotopic pattern. By bombarding the surface of a solid sample with an energetic beam of ions, we observed the release of a dye-Mg complex from the hydroxide structure. The TOF-SIMS spectrum of the LH/AC sample presented in Figure 3b shows an intense peak for the C_19_H_15_O_5_N_2_Mg^+^ ion, which clearly indicates that the AC dye interacted with Mg^2+^ ions in the sample. Although no characteristic peak for Al-azo chromophore interaction appeared in the TOF-SIMS spectrum, such an interaction cannot be excluded.

**Figure 4:**
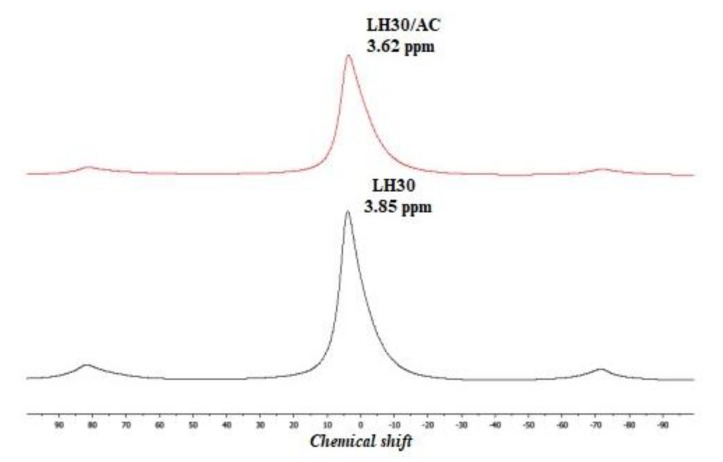
^27^Al solid-state nuclear magnetic resonance (MAS NMR) spectra of LH30 and LH30/AC samples.

The ^27^Al NMR technique was also employed to analyze the possible Al-dye complex. The ^27^Al spectrum of unmodified LH superimposed quite well onto the corresponding organic-inorganic phases. However, the spectrum for LH30/AC showed a peak at chemical shifts of 3.62 ppm, which can be assigned to Al in octahedral coordination (AlO_6_) (Figure 4) [34]. This suggests interaction between the chromophore and the LH material, which was sufficient to change the local environment of the Al atom slightly. Further comparison of the TOF-SIMS spectra for the pure LH30 samples and those modified with dye (Figure 3c) reveals that the addition of dye also resulted in a decrease in the emission of CO^2–^ ions. This change can be attributed to the lower concentration of CO_3_^2−^ in the LH30/AC sample, caused by their replacement in the LH matrix by dye anions. The observed decrease in CO_2_^−^ emission can therefore be taken as additional evidence of interactions between the azo dye molecules and metal ions from the LH matrix, as was observed in our previous works [32,35].

### 4.2. Powder X-ray Diffraction Analysis (PXRD)

**Figure 5:**
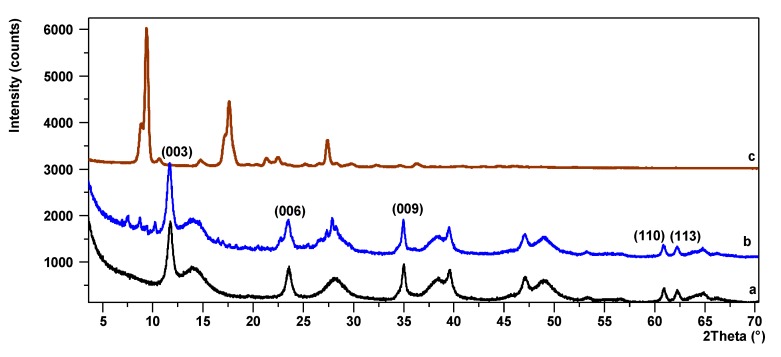
Powder X-ray diffraction (PXRD) patterns for LH20 (a), LH20/AC (b), AC (c).

**Figure 6:**
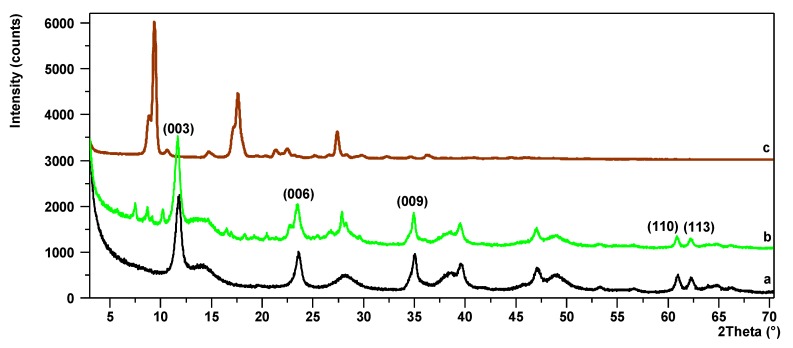
PXRD patterns for LH30 (a), LH30/AC (b), AC (c).

Powder X-ray diffraction was applied to investigate the influence of Mg/Al ratios on structural changes within the hybrid pigments. The PXRD diffraction patterns of LHs with different Mg/Al ratios (Figure 5a, Figure 6a and Figures S1a, S2a, LHs/AC (Figure 5b, Figure 6b and Figures S1b, S2b) and AC dye (are presented in the Figure 5c, Figure 6c and Figures S1c, S2c). The LH5 diffractogram (Figure S1a) shows short, wide peaks across the whole measuring range, indicating low crystallinity. The remaining diffraction patterns for LHs with Mg/Al ratios of 20/80, 30/70 and 70/30, show typical peaks for hydrotalcite materials with a layered structure [36]. The first two low-angle reflections are due to the diffraction on (003) and (006) planes and correspond to first and higher order diffraction respectively. On the slopes of the sharp peaks in the diffraction patterns of LH20 and LH30, the small broad peaks indicate the presence of co-precipitated poorly crystalline boehmite. After the modification, the basal reflections (003) of the LH/AC pigments (Figure 5c, Figure 6c and Figure S2c) are not shifted clearly into lower *2θ* angles, so the LH structures were generally retained. In all the diffraction patterns for LH/AC, a series of small reflections can be observed, probably belonging to the AC dye, as well as new peaks that do not match either the LH or the azo chromophore.

### 4.3. Morphology and Surface Investigation

The morphologies of the modified and unmodified LH powders were characterized by SEM and STEM techniques.

**Figure 7:**
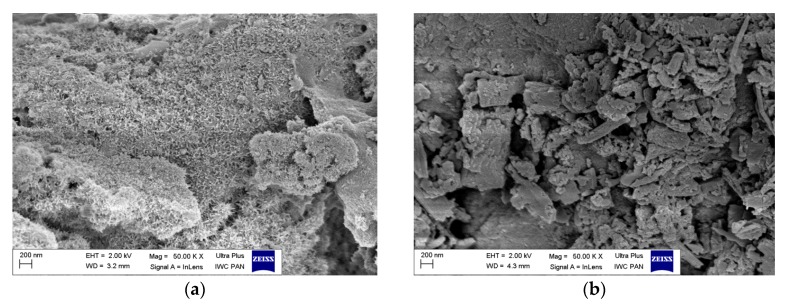

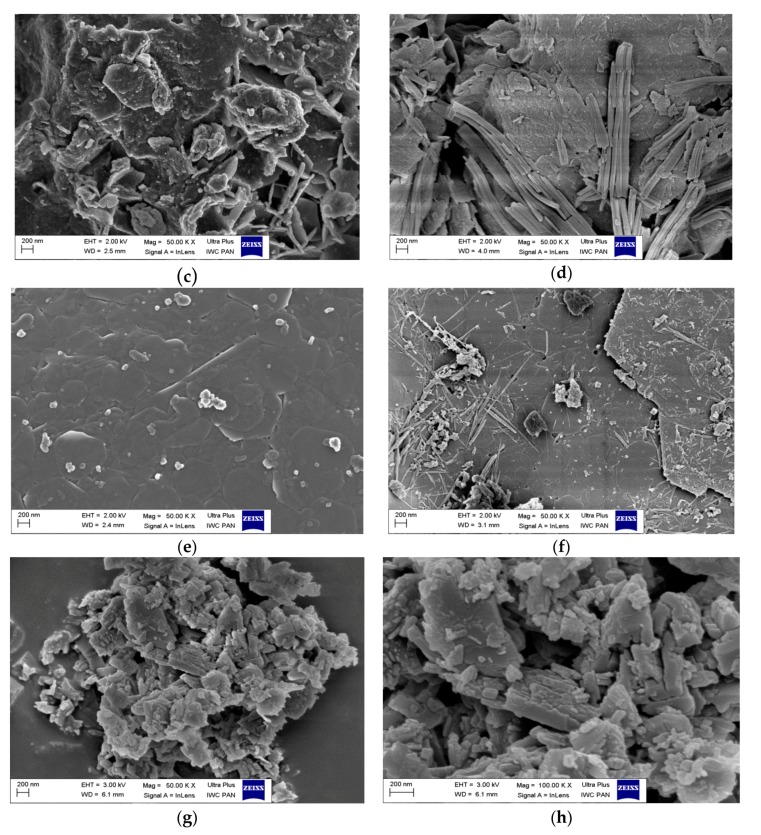
Scanning electron microscopy (SEM) images of LH5 (**a**), LH5/AC (**b**), LH30 (**c**), LH30/AC (**d**), LH70 (**e**), LH70/AC (**f**), and AC crystals (**g**,**h**).

**Figure 8:**
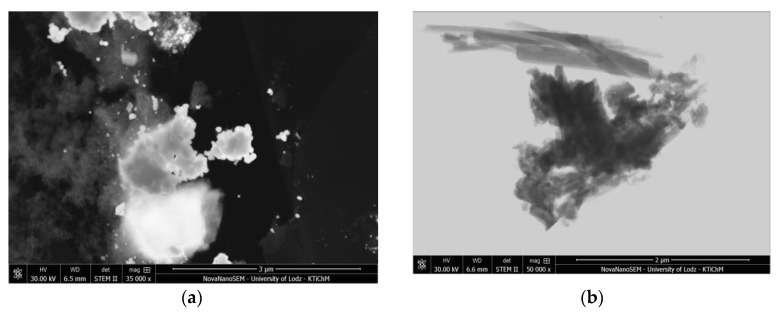

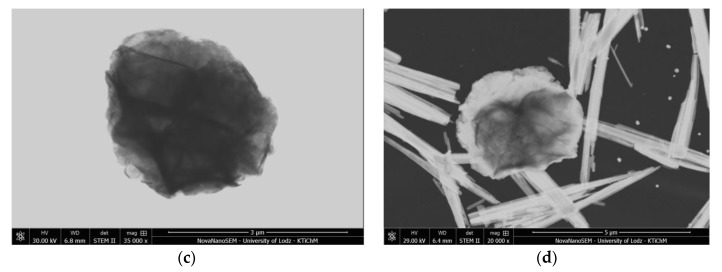
Scanning transmission electron microscopy (STEM) images of LH5 (**a**), LH5/AC (**b**), LH30 (**c**), and LH30/AC (**d**).

SEM and STEM microphotographs (Figure 7a and Figure 8b) show agglomerations of the raw LH5 (Mg/Al ratio is 5/95) particles ranging from 20 to 30 nm in size. The shape of the particles in the inorganic hosts changed gradually with increasing amounts of magnesium ions. As shown in Figure 7c, the morphology of the sample LH30 with higher concentrations of Mg ions exhibited numerous aggregated sheets, consisting of [Mg^2+^_1−x_Al^3+^_x_(OH)_2_]^x+^ layers connected to each other in irregular shapes. The lower concentration of the LH20 sample contributed to its larger surface area (230 m^2^/g) in comparison to LH30 (93 m^2^/g) and LH70 (17 m^2^/g), as determined by N_2_ measurements (Table 1). The Mg-ion-rich host (LH70) was characterized by lateral dimensions ranging from 500 nm in width to 10–50 nm in thickness. The Mg/Al ratio of this carrier resulted in a typical hydrotalcite-like structure (as confirmed by XRD study), characterized as a layered structure of uniform, hexagonal platelets lying flat on one another.

As can be seen from the SEM images (see Figure 7b,d,f,h and Figure 8b,d), the surface morphology of the hybrid pigments was substantially different from that of the unmodified LHs, due to incorporation of the azo dye into the inorganic structures. The micrographs revealed, in addition to the layers of carriers, the presence of dye crystals, which differed significantly in terms of shape and size from the original chromophore. This result was supported by the results of XRD studies and may be related to the creation of new organic-inorganic crystal forms, caused by the LH-dye interaction.

Interestingly, the final concentration of the incorporated dye depended on the concentration of magnesium ions and the surface area of the inorganic carriers. The effectiveness of the incorporation of the chromophore into the LH structures was assessed in terms of the amount of elemental nitrogen detected in the organic-inorganic pigments (Table 1).

As can be seen, the concentration of the dye molecules on the LH surface increased with the quantity of magnesium ions present in the LH hosts. However, despite the large amounts of magnesium ions in the LH70 structure, elemental analysis revealed that it had the lowest concentration of azo chromophore. The explanation for this may be the small specific surface area of the LH and the limited contact between the dye and LH matrix. It can be concluded that the mechanism of hybrid pigment formation is largely determined by the quantity of magnesium ions and their accessibility in the host structure.

### 4.4. Thermal Analysis and Solvent Resistance

**Figure 9:**
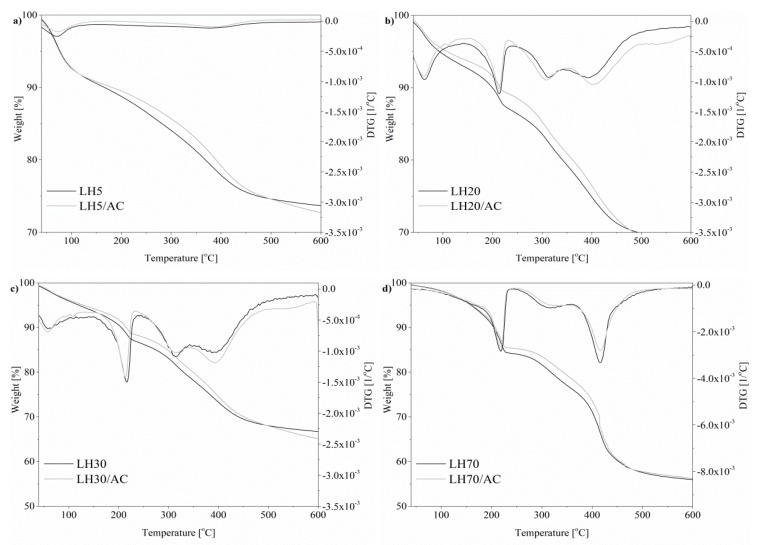
Thermogravimetric (TGA) and differential (DTG) curves for hybrid pigments based on different hosts: (**a**) LH5, (**b**) LH20, (**c**) LH30 and (**d**) LH70.

In Figure 9a–d and Table 2, TGA–DTA results obtained for aluminum-magnesium hydroxycarbonates before and after modification are presented.

Generally, for most of the samples with various Mg/Al ratios, the first mass loss step observed between ambient temperature and about 100 °C involved the loss of physically adsorbed water from LH structures (Figure 9a–c). Another weight loss took place at around 225 °C. This may be attributed to the dehydration of water molecules from interlayer space area. The very sharp third transition in the range 220–315 °C corresponds to the removal of hydroxyl groups from the brucite layers of LH host. The LH20, LH30 and LH70 samples also showed very broad, small peaks at around 334 °C (appearing before the main transition peak at 400 °C) which can be ascribed to the partial loss of OH^−^ in the brucite-like layers. The final step, occurring between 380 °C and 400 °C, may be attributed to the loss of hydroxyl groups and the decomposition of CO_3_^2–^ ions in the LH structure [37].

The similar shapes of the curves for LH20, LH30 and LH70 suggest similar structures (the XRD patterns of these LH hosts were also similar). The evident differences are only visible in terms of the total mass loss. However, the thermal stability of the LH carriers typically depended on the crystallinity and composition of the brucite-like layers. Magnesium-doped carrier LH5 revealed the lowest T_5%_ (77 °C) and was also characterized by the smallest degree of crystallization, as determined by XRD. The fact that LH70 showed the best thermal stability is most likely related to the large amount of magnesium in the sample, as well as to the increased structural ordering and greater crystallinity of the inorganic host.

**Figure 10:**
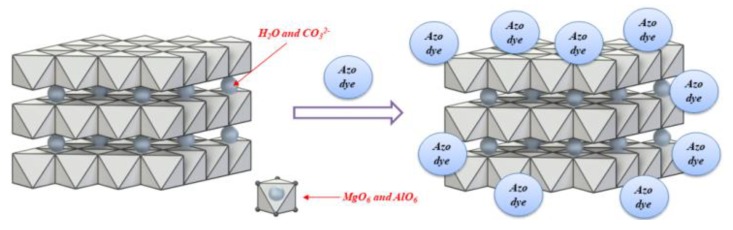
Proposed scheme of aluminum-magnesium hydroxycarbonate (when Mg/Al ratios are 20/80, 30/70 or 70/30) modified with azo dye.

After modification, the shapes of the thermograms and the positions of the peaks on the DTA curves were similar to those of the raw materials. However, in line with the results of our previous study [32,35], a slight increase in stability was observed for all the hybrid pigments, especially in the region above 300 °C. Two possible reasons for this may be proposed. First, the decomposition temperature of azo dye up to 250 °C is higher than that of LH (Figure S3). Second, the interaction between the dye and the LH matrix contributed to the decrease in the concentration of CO_3_^2−^ ions in the LH matrix, as was confirmed by TOF-SIMS. Therefore, the mechanism for the formation of the organic inorganic pigment with this chromophore is analogous to that proposed in our previous works (in which we used other azo and anthraquinone chromophores) [31,32], and is related mainly to interactions between acidic groups from the azo dye and the alkaline LH host (Figure 10).

**Figure 11:**
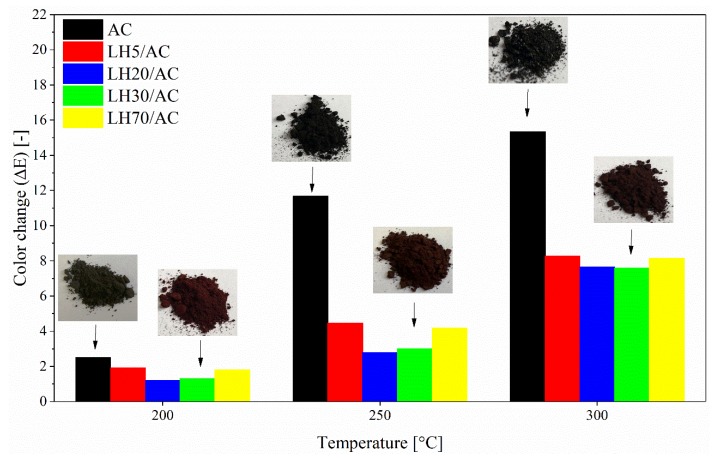
Total color difference (Δ*E*) values for AC dye and LH-based pigment at elevated temperatures.

The color stability of the prepared hybrids under extreme heat was investigated using spectrophotometric analysis in the CIE 1976 L*a*b* color space system. Figure 11 presents the total color change (Δ*E*) for samples at three different temperatures. The color change values of the powder samples become larger as the temperature increases, indicating a rise in the degree of thermal degradation. The color change was quite small, even at 300 °C, compared to the pure dye. The ΔE value of the azo dye changed from 2 to 12 when the temperature was boosted from 200 to 250 °C. This means that the pristine azo chromophore underwent thermal decomposition at much lower temperatures than the LH/AC hybrids. The reason for this may be the existence of strong host-guest interactions between the metal ions from the host matrix layers and azo chromophore guest anions.

The solvent resistance of the LH-based pigments was studied by immersing the samples in butyl acetate for 24 h (Figure S4). The azo chromophores were found to be soluble in the tested solvent, which turned an intense color, whereas the LH-based pigments exhibited high resistance to dissolution (the solvents remained colorless).

### 4.5. Fire Behavior

A cone calorimeter is a widely-used measurement tool for evaluating the flammability of composites under conditions resembling a real fire. In our study, a cone calorimeter was used to investigate the various impacts of the AC dye, LH hosts (with different Mg/Al weight ratios) and hybrid pigments on the flammability of ethylene-norbornene copolymer. The results obtained, including time to ignition (TTI), maximum peak of heat release rate (HRR_MAX_), maximum effective heat of combustion (EHC_MAX_), total heat release (THR) and mass loss rate (MLR), are presented in Table 3. As can be seen, the presence of AC dye and the combination of AC-LH can synergistically enhance the fire resistance of EN copolymer. In some systems, the application of hybrid pigments (EN/LH5/AC and EN/LH70/AC) was more effective than either unmodified LHs or AC dye in terms of improving flame retardance. This effect can be attributed to the stabilization of the azo chromophore on the LH host.

The azo dye, LH hosts, and above all, the hybrid pigments, all led to improvements in the fire resistance of EN copolymer, even at low filler concentrations (5 phr). Incorporation of the hybrid pigments and/or unmodified LH hosts effectively reduced the flammability of the EN composites, as evidenced by the decreases in the HRR_MAX_, EHC_MAX_ and THR parameters. For instance, the HRR_MAX_ parameter for neat EN copolymer was 427.8 kW/m^2^, while with LH-based pigments it decreased to around 375 (LH5/AC), 398 (LH20/AC), 385 (LH30/AC) or 330 (LH70/AC) kW/m^2^.

It was further noted that, in the initial phase of thermal decomposition, the heat transfer within the polymer matrix was limited, indicating a strong barrier effect within the LH-based materials. Azo dye incorporated on an LH carrier consists of condensed rings, which may form a protective carbon layer that acts as a “thermal shield”, effectively reflecting thermal radiation. The incorporation of inorganic fillers, such as clays or hydrotalcites, in a polymer matrix has been reported to enable the formation of a compact residue layer separating the flame region from the molten part which acts as protective layer, limiting heat transfer from the former region to the latter [38].

## 5. Conclusions

In this work, organic-inorganic pigments were prepared by precipitation of azo chromophore onto aluminum-magnesium hydroxycarbonates with various Mg/Al ratios. The ^27^Al MAS spectra of the organic-inorganic pigment revealed a chemical shift of the resonance peak in comparison to unmodified host, suggesting possible interactions of the studied dye with Al ions. Moreover, the TOF-SIMS spectrum of the LH modified with azo dye (AC) showed an intense peak for the C_19_H_15_O_5_N_2_Mg^+^ ion, indicating successful stabilization of azo chromophore molecules on the LH surface. The results of SEM and XRD confirmed the formation of new organic-inorganic crystal structures following LH modification. It was found that the concentration of the dye complexes depended on the level of magnesium ions and the surface area of the LH hosts. Improved solvent and thermal resistance of hybrid pigments arises from the strong interactions between the azo dye with the metal ions present in the inorganic sample. The flammability of the composites filled with organic-inorganic pigments was studied using the cone calorimeter test. The results for EN/hybrid pigment composites showed a lower values of the heat release rate than those for a neat sample (unfilled EN). Organic-inorganic pigments can thus play a dual role in EN composites, providing intense color and at the same time flame-retardant properties.

## Figures and Tables

**Table 1:** Results for surface area and N content from elemental analysis of studied hybrid pigments.

Sample	Specific Surface Area (m^2^/g)	%N
LH5	319	-
LH5/AC	207	0.745; 0.884; 0.818
LH20	230	-
LH20/AC	166	1.433; 1.427; 1.481
LH30	93	-
LH30/AC	87	1.622; 1.588; 1.566
LH70	17	-
LH70/AC	9	0.380; 0.410; 0.400

**Table 2:** Thermogravimetric analysis of aluminum-magnesium hydroxycarbonate (LH)-based pigments.

Sample	T_5%_ (°C)	T_10%_ (°C)	T_20%_ (°C)
LH5	77	166	368
LH5/AC	77	181	390
LH20	93	199	341
LH20/AC	109	216	362
LH30	120	206	330
LH30/AC	130	216	350
LH70	160	205	318
LH70/AC	161	208	342

T_5%_, T_10%_, T_20%_—Temperature at 5, 10 and 20% weight loss.

**Table 3:** Cone calorimeter results for neat ethylene-norbornene (EN) copolymer and compounds with AC dye, LH hosts and hybrid pigments.

Sample	TTI (s)	HRR_MAX_^1^ (kW/m^2^)	EHC_MAX_^2^ (MJ/kg)	THR ^3^ (MJ/m^2^)	MLR ^4^ (g/m^2^∙s)
EN	116	427.8 ± 8.6	79.0 ± 1.6	68.1 ± 1.4	6.90 ± 0.13
EN/AC	134	408.1 ± 8.1	69.9 ± 1.4	64.4 ± 1.3	6.40 ± 0.12
EN/LH5	80	380.5 ± 7.6	70.7 ± 1.4	62.1 ± 1.2	6.63 ± 0.13
EN/LH5/AC	120	375.7 ± 7.5	63.5 ± 1.2	61.6 ± 1.2	6.02 ± 0.12
EN/LH20	73	364.1 ± 7.2	65.3 ± 1.3	68.6 ± 1.4	6.83 ± 0.13
EN/LH20/AC	119	398.6 ± 7.9	67.6 ± 1.3	69.4 ± 1.5	6.65 ± 0.13
EN/LH30	82	362.9 ± 7.2	64.1 ± 1.2	64.5 ± 1.3	5.79 ± 0.11
EN/LH30/AC	105	385.6 ± 7.7	65.8 ± 1.3	65.2 ± 1.3	6.13 ± 0.12
EN/LH70	70	349.6 ± 6.9	65.7 ± 1.3	67.2 ± 1.4	6.10 ± 0.12
EN/LH70/AC	125	330.1 ± 6.6	62.9 ± 1.3	64.1 ± 1.3	5.98 ± 0.11

^1^ Heat release rate; ^2^ Effective heat of combustion; ^3^ Total heat release; ^4^ Mass loss rate.

## Supplementary Materials

The following are available online at https://www.mdpi.com/1996-1944/12/8/1349/s1, Figure S1: Powder XRD patterns for LH5 (a), LH5/AC (b), AC (c), Figure S2: Powder XRD patterns for LH70 (a), LH70/AC (b), AC (c), Figure S3: TGA/DTG curves of azo chromophore (AC),Figure S4: Digital images of AC chromophore (a), LH5/AC (b), LH20/AC (c), LH30/AC (d) and LH70/AC (e) after 24 h of immersion in butyl acetate.
