# Supplementary material for: New Organic/Inorganic Pigments Based on Azo Dye and Aluminum-Magnesium Hydroxycarbonates with Various Mg/Al Ratios
Source: Materials (Basel). 2019 Apr 25;12(8):1349. doi: 10.3390/ma12081349 (PMC6515457; doi:10.3390/ma12081349)
Supplement: Supplementary file 1 [file materials-12-01349-s001.pdf]

Supplementary Materials: New Organic/Inorganic Pigments based on Azo Dye and Aluminum-Magnesium Hydroxycarbonates with Various Mg/Al Ratios

Anna Marzec [1,*], Bolesław Szadkowski [1], Jacek Rogowski [2], Waldemar Maniukiewicz [2], Przemysław Rybiński [3] and Mirosława Prochoń [1]

[1] Institute of Polymer and Dye Technology, Faculty of Chemistry, Lodz University of Technology, Stefanowskiego 12/16, 90-924 Lodz, Poland; boleslaw.szadkowski@edu.p.lodz.pl (B.S.); miroslawa.prochon@p.lodz.pl (M.P.)
[2] Institute of General and Ecological Chemistry, Lodz University of Technology, Zeromskiego 116, 90-924 Lodz, Poland; jacek.rogowski@p.lodz.pl (J.R.); waldemar.maniukiewicz@p.lodz.pl (W.M.)
[3] Department of Management and Environmental Protection, Jan Kochanowski University, Kielce, Poland; przemyslaw.rybinski@ujk.edu.pl
* Correspondence: marzec.anna@hotmail.com

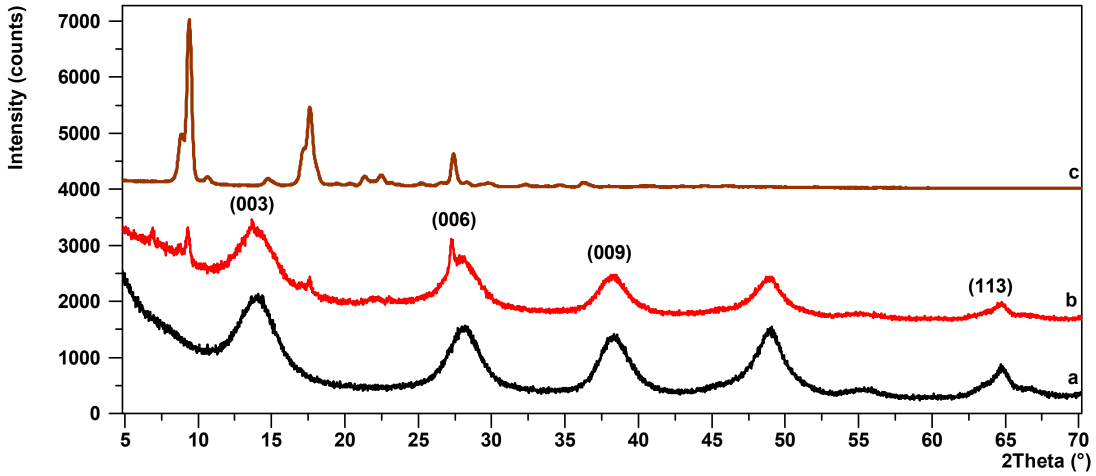

Figure 1. Powder XRD patterns for LH5 (a), LH5/AC (b), AC (c).

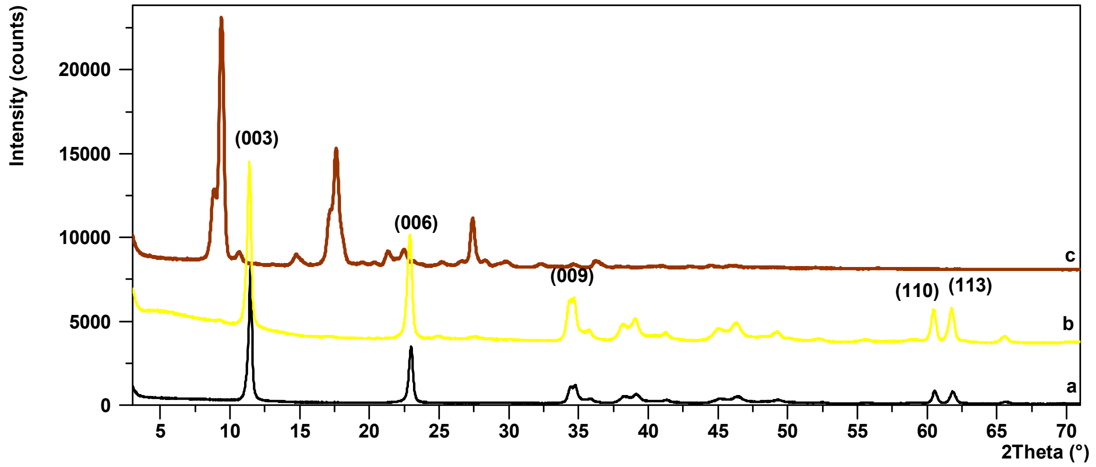

Figure 2. Powder XRD patterns for LH70 (a), LH70/AC (b), AC (c).

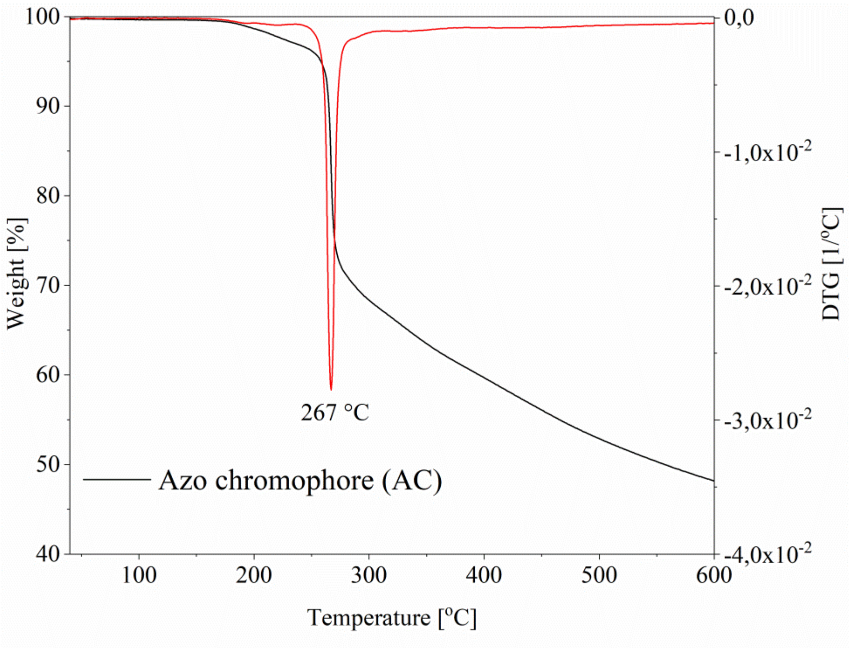

Figure 3. TGA/DTG curves of azo chromophore (AC).

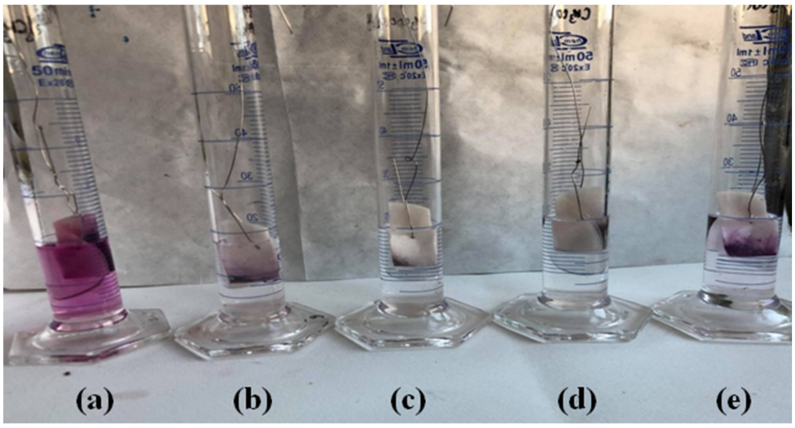

Figure 4. Digital images of AC chromophore (a), LH5/AC (b), LH20/AC (c), LH30/AC (d) and LH70/AC (e) after 24 h of immersion in butyl acetate.

© 2019 by the authors. Submitted for possible open access publication under the terms and conditions of the Creative Commons Attribution (CC-BY) license (http://creativecommons.org/licenses/by/4.0/).
